# The Effect of Cross-Linking with Citric Acid on the Properties of Agar/Fish Gelatin Films

**DOI:** 10.3390/polym12020291

**Published:** 2020-02-02

**Authors:** Jone Uranga, Bach T. Nguyen, Trung Trang Si, Pedro Guerrero, Koro de la Caba

**Affiliations:** 1 BIOMAT Research Group, University of the Basque Country (UPV/EHU), Escuela de Ingeniería de Gipuzkoa, Plaza de Europa 1, 20018 Donostia-San Sebastián, Spain; 2 Faculty of Food Technology, Nha Trang University, 02 Nguyen Dinh Chieu Street, Nha Trang City 650000, Vietnam; ntbachnt@ntu.edu.vn (B.T.N.); trungts@ntu.edu.vn (T.T.S.)

**Keywords:** fish gelatin, agar, citric acid, cross-linking

## Abstract

The aim of this work was to assess the effect of fish gelatin–citric acid nucleophilic substitution and agar–citric acid esterification reactions on the properties of agar/fish gelatin films. Since temperature is an important cross-linking parameter, films were treated at 90 °C and 105 °C and film properties were compared to those of non-cured films. It was observed that temperature favored the aforementioned reactions, which induced physical and morphological changes. In this regard, darker films with a rougher surface were obtained for the films with a higher cross-linking degree. While mechanical properties were slightly modified, the barrier properties were enhanced due to the reactions that occurred. Therefore, these agar/fish gelatin films cross-linked through two different reactions can be considered to be promising materials as active films for different purposes, such as active packaging or pharmaceutical applications.

## 1. Introduction

The valorization of biowastes and their utilization as raw materials leads to a more efficient use of resources and promotes a more circular economy [1,2]. Biopolymers obtained from biowastes can be employed to prepare novel materials for a wide range of purposes, such as food packaging, biomedical, and cosmetic applications [3,4,5]. In addition to biopolymers, additives that are capable of improving the functional properties and versatility of biopolymers can also be obtained from biowastes [6,7].

With regard to food processing wastes, fishery residues are promising candidates from which to extract biopolymers and bioactives. According to recent findings, fishery discards account for slightly less than 10% of total annual catches, which correspond approximately to 10 Mt/year [8]. Furthermore, fish processing wastes include large quantities of substandard muscles, viscera, heads, skins, fins, frames, and trimmings, which generally account for 30–50% of the total weight of the starting material [9]. These fish wastes represent a source of several potentially valuable molecules [2]—among them, gelatin [10], a protein with unique functional and technological properties. This protein is an easily processable material due to its linear structure and limited monomer composition, leading to excellent film forming properties [11]. However, gelatin is moisture-sensitive [12,13] and has weak mechanical properties [14]; therefore, some modifications are necessary to enhance the mechanical behaviour and water stability of gelatin-based materials and, thus, to extend their application domain [15].

Besides fishery residues, other biowastes that can give rise to diverse environmental issues are generated in nature. For instance, marine algae are washed ashore on beaches and they can alter the sedimentary balance along the overall transverse beach profile [16]. In order to prevent possible problems, this biowaste can be valorized to obtain agar, a fibrous polysaccharide that consists of two main components: agarose and agaropectin [17]. This hydrophilic colloid is extracted from marine algae of the *Rhodophyceae* class, such as *Gelidium* sp. and *Gracilaria* sp. [18,19]. Agar has shown the ability to form very hard gels at very low concentrations and, thus, is widely used in food and pharmaceutical industries as gelling and thickening agents [20]. This polysaccharide has excellent film forming abilities, thermoplasticity, biocompatibility, and moderate water resistance [21].

In this context, different cross-linkers, such as citric acid [22], have been employed towards the enhancement of the biopolymer’s functional properties. In this regard, citric acid is biocompatible, water soluble, non-toxic, and inexpensive [23,24,25]. This mild organic acid is a polycarboxylic acid that occurs naturally in citrus fruits [26,27]. The cross-linking of polysaccharides with citric acid occurs mainly through the hydroxyl groups of the polysaccharide and the carboxyl groups of the acid; it is known as an esterification reaction [28]. In regards to the cross-linking of proteins, the mechanism lies in the nucleophilic substitution falling between the carboxyl groups of the acid and the amino groups of the protein, forming stable amide bonds [29].

Taking the above into consideration, the aim of this work was to cross-link fish gelatin and agar with citric acid in order to obtain agar/fish gelatin films with improved properties. Even if agar/gelatin blends have been previously analyzed, this is the first study—to the best of our knowledge—that deals with the cross-linking of both polymers with citric acid. Furthermore, since temperature is known to be an important cross-linking parameter, different thermal treatments were compared and the physicochemical, mechanical, morphological, and optical properties were assessed.

## 2. Materials and Methods

### 2.1. Materials

The gelatin from fish skin (*Pangasius hypopthalmus*), with 250 bloom and 11% moisture content, was purchased from Vinh Hoan Collagen’s Factory (Dong Thap province, Vietnam). Agar, with 12% moisture content and 4.63 g SO_4_^2−^/kg, was provided by Hai Long Robika Factory (Dong Thap province, Vietnam). Glycerol, with a purity of 99.01%, and anhydrous citric acid were obtained from Panreac (Barcelona, Spain). All chemicals were of food grade and they were used as received without further purification.

### 2.2. Preparation of Films

The agar/fish gelatin films were prepared by solution casting. First, 5 g of fish gelatin was mixed with citric acid (30 wt % on a gelatin basis) and 60 mL of distilled water at 80 °C and 200 rpm for 30 min, while the agar (40 wt % on a gelatin basis) was dissolved in 40 mL of distilled water at 110 °C and 200 rpm for 30 min. Then, these two solutions were mixed, 20 wt % glycerol (on a gelatin basis) was added as a plasticizer, and the mixture’s pH was adjusted to pH 10 with NaOH (1 M). The agar/fish gelatin solution was heated at 80 °C and 200 rpm for 30 min. Finally, the solution was poured into Petri dishes and left to dry for 48 h at room temperature to obtain the agar/fish gelatin films. Some films were subjected to heating at 90 or 105 °C for 24 h. Non-heated films were designated as control films, while thermally treated films were designated as 90 °C and 105 °C samples as a function of the employed temperature. All films were conditioned at 25 °C and 50% relative humidity in a climatic chamber before testing.

### 2.3. Moisture Content, Swelling, and Hydrolytic Degradation Tests

The films were weighed (w_0_) and then dried in an oven at 105 °C for 24 h. After this time, the samples were reweighed (w_1_) to determine moisture content (MC):

$$\mathrm{MC}\;(\%)=\frac{w_{0}-w_{1}}{w_{0}}\;100.$$

Then, the films were immersed into a phosphate-buffered saline (PBS) solution at room temperature and weighed after immersion into the PBS solution for specific times (w_t_), until it reached a constant value. Swelling (SW) was calculated according to the following equation:

$$\mathrm{SW}\;(\%)=\frac{w_{t}-w_{1}}{w_{1}}\;100.$$

Once the swelling test ended, the samples were removed from the PBS, wiped with a paper, left to dry at room temperature for 24 h, and reweighed (w_f_) to determine their hydrolytic degradation degree (DD):

$$\mathrm{DD}\;(\%)=\frac{w_{1}-w_{f}}{w_{1}}\;100.$$

### 2.4. Cross-Linking Extent

The fish gelatin cross-linking extent was measured according to the method of Panzavolta et al. [30]. Briefly, an UV assay of uncross-linked amino groups was performed on thermally treated films and on non-cured films as a reference. After the reaction with 0.5% 2,4,6-trinitrobenzenesulfonic acid (TNBS), the gelatin was hydrolyzed with HCl (6 M) and extracted with diethyl ether. The solution absorbance was measured against a blank by UV-vis spectroscopy at a wavelength of 346 nm. The moles of free amino groups per gram of gelatin were calculated by the following equation:

$$\left[{\mathrm{NH}}_{2}\right]=\frac{2\cdot A\cdot V}{\varepsilon \cdot b\cdot x}$$

where A is the sample absorbance, V is the final sample volume (L), Ɛ is the molar absorptivity of TNP-lys (precisely 1.46 × 104 L·mol^−1^·cm^−1^), b is the cell path length (cm), and x is the sample weight (g).

The cross-linking extent was determined from the ratio between the moles of cross-linked amino groups of thermally treated films (obtained as the difference between uncross-linked groups before and after cross-linking) with respect to the amino groups measured in the non-cured film.

### 2.5. Fourier Transform Infrared (FTIR) Spectroscopy

FTIR analysis of films was carried out on a Nicolet 380 FTIR spectrometer (Nicolet Instrument, Barcelona, Spain) using an ATR Golden Gate. A total of 32 scans were performed at a resolution of 4 cm^−1^ in the wavenumber range from 4000 to 800 cm^−1^.

### 2.6. Tensile Test

Tensile strength (TS) and elongation at break (EB) were determined using an Insight 10 Electromechanical Testing System (MTS Systems, Madrid, Spain), equipped with a tensile load cell of 250 N. According to ASTM D638-03, the crosshead speed was set at 1 mm/min, and samples with a length of 22.25 mm and a width of 4.75 mm were used.

### 2.7. Scanning Electron Microscopy (SEM)

The film’s inner and surface morphology was visualized using a Hitachi S-4800 field emission scanning electron microscope (Hitachi High-Technologies Corporation, Madrid, Spain). Samples were mounted on a metal stub with a double-side adhesive tape and coated under vacuum with gold (JFC-1100) in an argon atmosphere prior to observation. All samples were examined employing an accelerating voltage of 10 kV.

### 2.8. X-ray Diffraction (XRD)

XRD analysis was performed with a diffraction unit (PANalytic Xpert PRO, Madrid, Spain) operating at 40 kV and 40 mA. The radiation was generated from a Cu-Kα (λ = 1.5418 Å) source. The diffraction data were obtained from 2θ values from 2° to 50°, where θ is the incidence angle of the X-ray beam on the sample.

### 2.9. Color Measurement

Color parameters (L*, a*, b*) were determined using a CR-400 Minolta Chroma-Meter colorimeter (Konica Minolta, Valencia, Spain). Films were placed on the surface of a white standard plate (calibration plate values: L* = 97.39, a* = 0.03, b* = 1.77) and color parameters were measured using the CIELAB color scale: L* = 0 (black) to L* = 100 (white), −a* (greenness) to +a* (redness), and −b* (blueness) to +b* (yellowness). The color difference (ΔE*) was calculated with reference to the non-cured film:

$$\Delta E^{*}=\sqrt{{\left(\Delta L^{*}\right)}^{2}+{\left(\Delta a^{*}\right)}^{2}+{\left(\Delta b^{*}\right)}^{2}}.$$

### 2.10. Gloss Measurement

Film gloss was determined using a Multi Gloss 268 Plus gloss meter (Konica Minolta, Valencia, Spain). Gloss values were measured at a 60° incidence angle, according to ASTM D523-14.

### 2.11. UV-Vis Spectroscopy

Light absorption was measured in the UV-vis range (200–800 nm) using a V-630 UV-vis spectrophotometer (Jasco, Barcelona, Spain).

### 2.12. Statistical Analysis

Analysis of variance (ANOVA) was used to determine the significance of differences among samples. The analysis was performed with a SPSS computer program (SPSS Statistic 23.0, IBM, Armonk, NY, USA) and Tukey’s test was used for multiple comparisons. The differences were statistically significant at the *p* < 0.05 level. A minimum of five samples were analyzed.

## 3. Results and Discussion

### 3.1. Physicochemical Properties

Since low MC values are desirable for some applications in order to prevent the microorganisms’ growth, the MC of films was measured, and the values are shown in Table 1. MC values were around 12% for control films and slightly (*p* < 0.05) lower for thermally treated films due to moisture evaporation during film heating—although moisture was absorbed again during conditioning, reaching a MC value of around 11%. The heating temperature did not significantly (*p* > 0.05) affect MC values.

**Figure 1:**
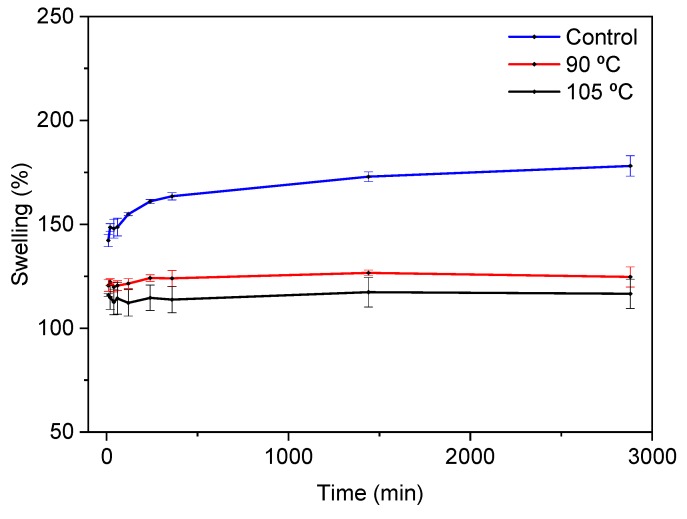
Swelling behaviour of control and thermally treated films.

In a similar way, control films showed the highest swelling values (Figure 1) as well as the highest hydrolytic degradation degree (50%), which was significantly (*p* < 0.05) decreased for thermally treated films, with DD values of 26% and 22% for the films heated at 90 °C and 105 °C, respectively. The swelling behaviour observed and the DD values measured are indicative of cross-linking and, thus, the cross-linking extent was calculated. The cross-linking extent values confirmed the effect of temperature, since the cross-linking degree significantly (*p* < 0.05) increased from 45 ± 4% for the films heated at 90 °C to 67 ± 7% for the films heated at 105 °C.

**Figure 2:**
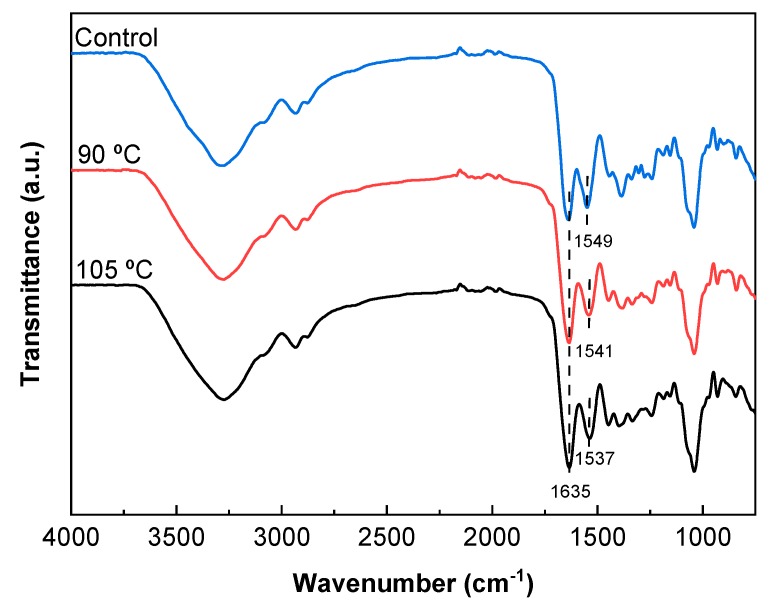
FTIR spectra of control and thermally treated films.

In order to further assess physicochemical interactions among the components of the film forming formulation, FTIR analysis was carried out. As seen in Figure 2, all films showed the broad absorption band characteristic at 3500–3000 cm^−1^. This can be attributed to the free and bounded -NH groups of gelatin and -OH groups of both gelatin and agar that were able to form hydrogen bonds [31].

Since pure agar does not have characteristic bands at around 1500–1700 cm^−1^ [32], the two bands that arose in this area were related to fish gelatin, which is associated with amide I (C=O stretching) and amide II (N-H bending). The amide I band at 1635 cm^−1^ did not change, while the amide II band shifted from 1549 cm^−1^ for control films to 1537 cm^−1^ for the films treated at 105 °C. This shift confirms the fact that the temperature promoted cross-linking, as previously shown by the cross-linking degree values. Moreover, there was no presence of the two characteristic bands of citric acid at 1690 cm^−1^ and 1743 cm^−1^ assigned to the C=O stretching of citric acid [22]. This fact suggested the cross-linking of citric acid with the biopolymers used in the film forming formulation. In this regard, agar molecules form double helices at low temperatures (30–40 °C), but they exist as random coils when dissolved in water at high temperatures (above 85 °C) [33]; therefore, the temperatures (80–110 °C) employed to prepare films in this work made the agar chains more accessible when reacting with citric acid by an esterification reaction. An almost inappreciable shoulder, located at around 1740 cm^−1^, can be related to the residual citric acid [34]. Such an esterification would improve the water resistance of films [35], as shown by the swelling curves in Figure 1. Furthermore, since the films were prepared at basic pH, the nucleophilic substitution between carboxylate groups in citric acid and amine groups in gelatin was favored [36].

### 3.2. Mechanical and Morphological Properties

**Figure 3:**
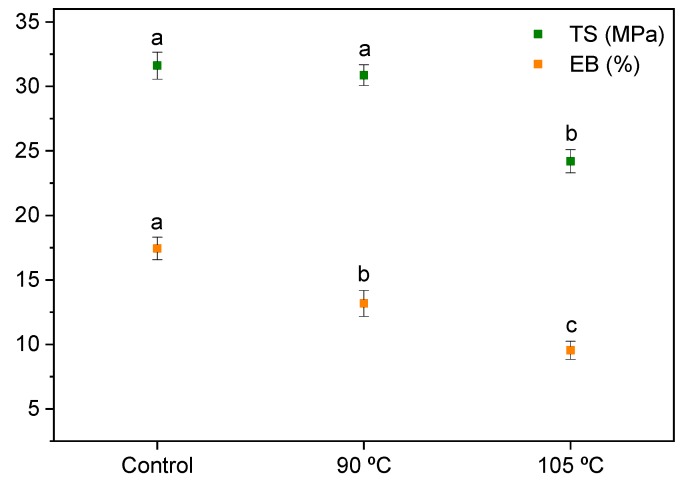
Tensile strength (TS) and elongation at break (EB) of control and thermally treated films. Two means followed by the same letter for the same color are not significantly (*p* > 0.05) different through Tukey’s multiple range test.

Tensile tests were carried out for agar/fish gelatin films, and the TS and EB values are shown in Figure 3. As seen here, there was no significant (*p* > 0.05) change of TS values between the control films and those heated at 90 °C, while EB decreased (*p* < 0.05) for the thermally treated films due to the cross-linking reactions of the biopolymers with citric acid, which led to a higher cross-linking extent for the films heated at 105 °C, as previously shown. Comparing these results with the properties of other agar/gelatin films [32], the cross-linking reactions with citric acid—and thus the increasing structural cohesion—resulted in the reduction of the film’s flexibility and the decrease of the EB values.

**Figure 4:**
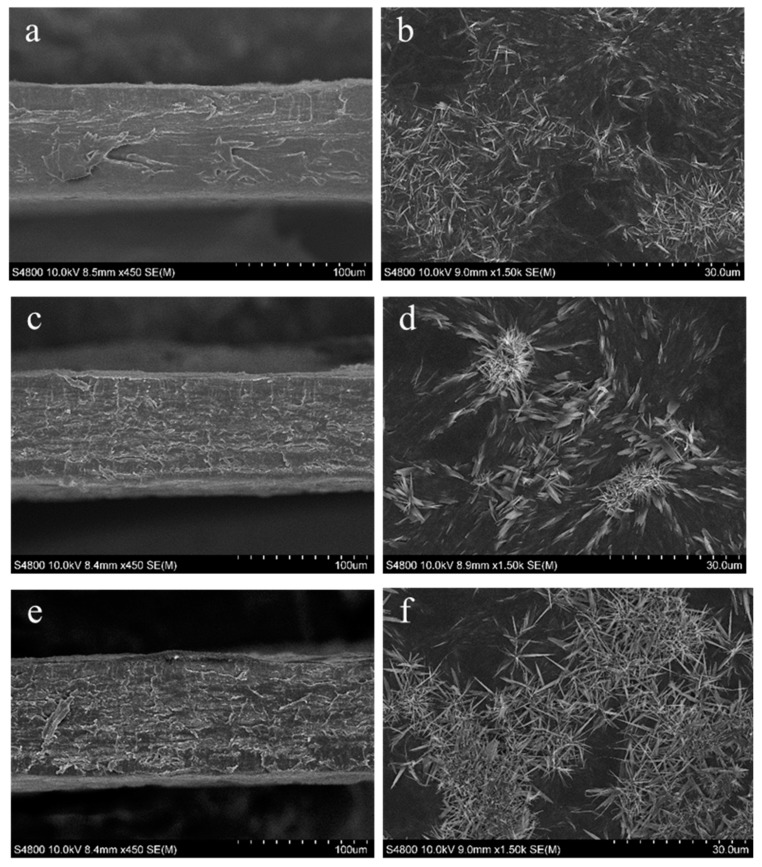
SEM images of cross-section (left hand) and surface (right hand) of control (**a**,**b**) and thermally treated films at 90 °C (**c**,**d**) and 105 °C (**e**,**f**).

In order to relate mechanical properties and film structure, SEM analysis was carried out (Figure 4). Overall, the films were compact and homogeneous. In fact, both the fish gelatin and agar chains have hydroxyl groups available for intermolecular interactions by hydrogen bonding, which improved the miscibility of the two phases [32]. Control films showed a smooth cross-section, while thermally treated films presented rougher cross-sections, probably due to the interactions promoted by heating. Roughness is an important factor for cell adhesion, proliferation, and differentiation [37], and since a higher temperature of curing (105 °C) led to rougher cross-sections, this temperature could be considered more appropriate for preparing materials for biomedical applications. Additionally, microcrystals were observed on the film’s surface. These microcrystals are typically observed in agar-containing materials [38,39].

**Figure 5:**
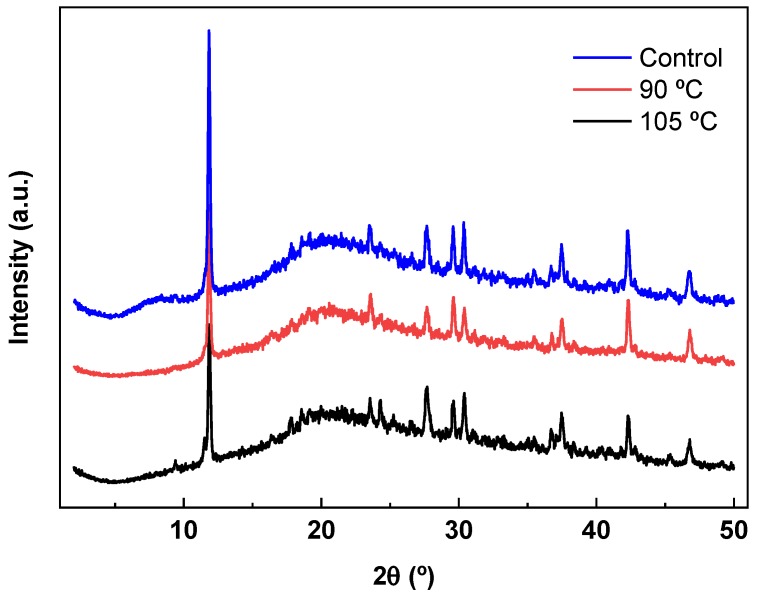
XRD patterns of control and thermally treated films.

The crystalline/amorphous nature of agar/fish gelatin films was also analyzed by XRD and the results are shown in Figure 5. The XRD pattern of the control film exhibited a distinctive diffraction peak at a 2θ value around 8°, corresponding to the triple-helix diameter of gelatin [40]. This peak disappeared for the thermally treated films, indicating the effect of heating in the film structure. This is in accordance with the decrease observed for TS values, indicative of the loss of the triple-helix structure after heating. The other characteristic peak of gelatin, located at about 21°, and the typical peak of agar, centred at about 20°, joined and became a wide band in all XRD patterns [41,42]. Moreover, an intense narrow peak—a characteristic of agar [43]—appeared at around 12°, indicating some crystallinity in agar/fish gelatin films, as also shown by the SEM analysis.

### 3.3. Optical Properties

Optical properties were also influenced by the interactions between fish gelatin, agar, and citric acid. Color and gloss values were determined and are shown in Table 2. Thermally treated films were darker, redder, and yellower (*p* < 0.05), and this increase with regards to the control film was significantly (*p* < 0.05) higher for the films heated at 105 °C, which showed the lowest L* values and the highest a*, b*, and ΔE* parameters. The yellow appearance is believed to occur due to the dehydration of citric acid, producing a colored, unsaturated acid after heating [29].

The films did not show glossy surfaces, with the gloss values lower than 10 GU. The control films were glossier than those that were thermally treated (*p* < 0.05), and the temperature employed did not significantly (*p* > 0.05) affect the gloss values. Since gloss is related to surface roughness, with higher values indicating smoother surfaces [44], the decrease of gloss values for thermally treated films indicated the formation of rougher surfaces, as also shown by SEM images.

**Figure 6:**
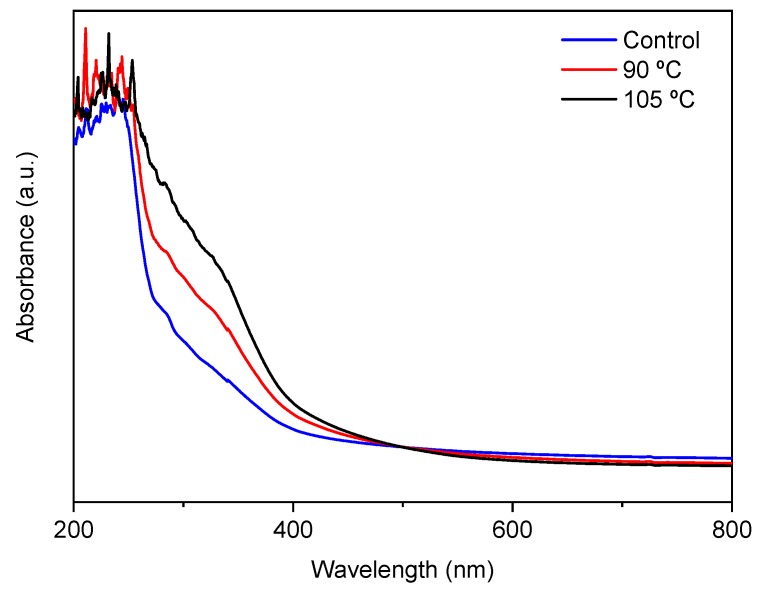
UV-vis spectra of control and thermally treated films.

Finally, UV-vis spectroscopy was employed in order to analyze the film’s light absorption. As seen in Figure 6, all films revealed a high absorption of UV light in the range of 200–250 nm. This might be due to the high content of tyrosine and phenylalanine, which are sensitive chromophores in fish gelatin and absorb light below 300 nm, playing an important role in UV barrier properties [45]. Moreover, pure agar has an absorption peak of around 221 nm due to the electronic transitions of agar [46], which would also contribute to the UV absorption of films. Furthermore, UV-vis spectra presented a particular absorption in the range of 250–400 nm, especially for thermally treated films, which was probably due to the formation of new compounds resulting from the cross-linking reactions mentioned above. This led to a higher UV absorption, preventing possible deterioration processes caused by UV light. Therefore, citric-acid-incorporated agar/fish gelatin films showed better UV barrier properties compared to conventional agar/gelatin films. This improvement could be interesting for food packaging applications, avoiding the food oxidative deterioration that leads to nutrient losses, discoloration, and off-flavors.

## 4. Conclusions

The cross-linking of fish gelatin and agar with citric acid was found to be an alternative way of enhancing the properties of agar/fish gelatin films. Cross-linking reactions were confirmed by FTIR and favored by heating, as shown in the cross-linking degree values and swelling curves. Furthermore, these cross-linking reactions led to the enhancement of agar/fish gelatin functional properties, such as UV light barrier properties and rough surfaces, as shown by SEM images and gloss values. Overall, resistant and easy-to-handle films based on biopolymers derived from marine sources were obtained, indicating the potential for natural and renewable resources to develop more sustainable products.

## Figures and Tables

**Table 1:** Moisture content (MC) of control and thermally treated films.

Film	MC (%)
Control	12.05 ± 0.36 ^a^
90 °C	11.23 ± 0.28 ^b^
105 °C	11.22 ± 0.09 ^b^

^a,b^ Two means followed by the same letter in the same column are not significantly (*p* > 0.05) different through Tukey’s multiple range test.

**Table 2:** Colour and gloss values of control and thermally treated films.

Film	L*	a*	b*	ΔE*	Gloss (GU)
Control	95.3 ± 0.3 ^a^	−0.8 ± 0.0 ^a^	11.9 ± 1.1 ^a^	-	8.9 ± 0.9 ^a^
90 °C	87.0 ± 0.8 ^b^	2.2 ± 0.3 ^b^	32.7 ± 0.8 ^b^	22.6 ± 1.0 ^a^	6.9 ± 0.9 ^b^
105 °C	82.8 ± 1.5 ^c^	5.1 ± 0.9 ^c^	44.3 ± 2.0 ^c^	35.2 ± 2.6 ^b^	6.5 ± 1.1 ^b^

^a–c^ Two means followed by the same letter in the same column are not significantly (*p* > 0.05) different through Tukey’s multiple range test.

## References

[1] De Clercq D., Wen Z., Fan F. (2017). Performance evaluation of restaurant food waste and biowaste to biogas pilot projects in China and implications for national policy. J. Environ. Manag..

[2] Xu C., Nasrollahzadeh M., Selva M., Issaabadi Z., Luque R. (2019). Waste-to-wealth: Biowaste valorization into valuable bio (nano) materials. Chem. Soc. Rev..

[3] Celli A., Colonna M., Gandini A., Gioia C., Lacerda T.M., Vannini M., Cavani F., Albonetti S., Basile F., Gandini A. (2016). Polymers from monomers derived from biomass. Chemicals and Fuels from Bio-Based Building Blocks.

[4] Alemán A., González F., Arancibia M., López-Caballero M.E., Montero P., Gómez-Guillén M.C. (2018). Development of active biocomposites using a shrimp cooking effluent. Innov. Food Sci. Emerg. Technol..

[5] Ma Q., Pang K., Wang K., Huang S., Ding B., Duan Y., Zhang J. (2019). Ultrafine and carboxylated β-chitin nanofibers prepared from squid pen and its transparent hydrogels. Carbohydr. Polym..

[6] Garrido T., Leceta I., de la Caba K., Guerrero P. (2018). Chicken feathers as a natural source of sulphur to develop sustainable protein films with enhanced properties. Int. J. Biol. Macromol..

[7] Saeb M.R., Rastin H., Nonahal M., Paran S.M.R., Khonakdar H.A., Puglia D. (2018). Cure kinetics of epoxy/chicken eggshell biowaste composites: Isothermal calorimetric and chemorheological analyses. Prog. Org. Coat..

[8] Zeller D., Cashion T., Palomares M., Pauly D. (2018). Global marine fisheries discards: A synthesis of reconstructed data. Fish Fish..

[9] Abejón R., Belleville M.P., Sanchez-Marcano J., Garea A., Irabien A. (2018). Optimal design of industrial scale continuous process for fractionation by membrane technologies of protein hydrolysate derived from fish wastes. Sep. Purif. Technol..

[10] Santos T.M., Filho M.M.S., Caceres C.A., Rosa M.F., Morais J.P.S., Pinto A.M.B., Azeredo H.M.C. (2014). Fish gelatin films as affected by cellulose whiskers and sonication. Food Hydrocoll..

[11] Weng W., Zheng H. (2015). Effect of transglutaminase on properties of tilapia scale gelatin films incorporated with soy protein isolate. Food Chem..

[12] Hosseini S.F., Gómez-Guillén M.C. (2018). A state-of-the-art review on the elaboration of fish gelatin as bioactive packaging: Special emphasis on nanotechnology-based approaches. Trends Food Sci. Technol..

[13] Kchaou H., Benbettaïeb N., Jridi M., Abdelhedi O., Karbowiak T., Brachais C.H., Léonard M.L., Debeaufort F., Nasri M. (2018). Enhancement of structural, functional and antioxidant properties of fish gelatin films using Maillard reactions. Food Hydrocoll..

[14] Chin S.S., Lyn F.H., Hanani Z.A.N. (2017). Effect of *Aloe vera* (*Aloe barbadensis* Miller) gel on the physical and functional properties of fish gelatin films as active packaging. Food Packag. Shelf Life.

[15] Zhao X., Zhou Y., Zhao L., Chen L., He Y., Yang H. (2019). Vacuum impregnation of fish gelatin combined with grape seed extract inhibits protein oxidation and degradation of chilled tilapia fillets. Food Chem..

[16] Bunicontro M.P., Marcomini S.C., Casas G.N., Makowski C., Finkl C.W. (2019). Environmental impacts of an Alien Kelp species (*Undaria pinnatifida*, Laminariales) along the Patagonian coasts. Impacts of Invasive Species on Coastal Environments.

[17] Jumaidin R., Sapuan S.M., Jawaid M., Ishak M.R., Sahari J. (2017). Thermal, mechanical, and physical properties of seaweed/sugar palm fibre reinforced thermoplastic sugar palm starch/agar hybrid composites. Int. J. Biol. Macromol..

[18] Atef M., Rezaei M., Behrooz R. (2014). Preparation and characterization agar-based nanocomposite film reinforced by nanocrystalline cellulose. Int. J. Biol. Macromol..

[19] Giménez B., de Lacey A.L., Pérez-Santín E., López-Caballero M.E., Montero P. (2013). Release of active compounds from agar and agar-gelatin films with green tea extract. Food Hydrocoll..

[20] Rodríguez-Félix A., Madera-Santana T.J., Freile-Pelegrín Y., Masuelli M.A. (2017). Formulation, Properties and Performance of Edible Films and Coatings from Marine Sources in Vegetable and Fruits. Biopackaging.

[21] Wang X., Guo C., Hao W., Ullah N., Chen L., Li Z., Feng X. (2018). Development and characterization of agar-based edible films reinforced with nano-bacterial cellulose. Int. J. Biol. Macromol..

[22] Uranga J., Leceta I., Etxabide A., Guerrero P., de la Caba K. (2016). Cross-linking of fish gelatins to develop sustainable films with enhanced properties. Eur. Polym. J..

[23] Naeini A.T., Adeli M., Vossoughi M. (2010). Poly (citric acid)-*block*-poly (ethylene glycol) copolymers—New biocompatible hybrid materials for nanomedicine. Nanomed. Nanotechnol. Biol. Med..

[24] Reddy N., Jiang Q., Yang Y. (2012). Preparation and properties of peanut protein films crosslinked with citric acid. Ind. Crops Prod..

[25] Tan S., Ebrahimi A., Langrish T. (2017). Template-directed flower-like lactose with micro-meso-macroporous structure. Mater. Des..

[26] Passos C.P., Ferreira S.S., Serôdio A., Basil E., Marková L., Kukurová K., Ciesarová Z., Coimbra M.A. (2018). Pectic polysaccharides as an acrylamide mitigation strategy—Competition between reducing sugars and sugar acids. Food Hydrocoll..

[27] Cumming M.H., Leonard A.R., LeCorre-Bordes D.S., Hofman K. (2018). Intra-fibrillar citric acid crosslinking of marine collagen electrospun nanofibres. Int. J. Biol. Macromol..

[28] Awadhiya A., Kumar D., Verma V. (2016). Crosslinking of agarose bioplastic using citric acid. Carbohydr. Polym..

[29] Liguori A., Uranga J., Panzavolta S., Guerrero P., de la Caba K., Focarete M.L. (2019). Electrospinning of fish gelatin solution containing citric acid: An environmentally friendly approach to prepare crosslinked gelatin fibers. Materials.

[30] Panzavolta S., Gioffrè M., Focarete M.L., Gualandi C., Foroni L., Bigi A. (2011). Electrospun gelatin nanofibers: Optimization of genipin cross-linking to preserve fiber morphology after exposure to water. Acta Biomater..

[31] Guerrero P., Garrido T., Leceta I., de la Caba K. (2013). Films based on proteins and polysaccharides: Preparation and physical-chemical characterization. Eur. Polym. J..

[32] Mohajer S., Rezaei M., Hosseini S.F. (2017). Physico-chemical and microstructural properties of fish gelatin/agar bio-based blend films. Carbohydr. Polym..

[33] Wang Y., Dong M., Guo M., Wang X., Zhou J., Lei J., Guo C., Qin C. (2017). Agar/gelatin bilayer gel matrix fabricated by simple thermo-responsive sol-gel transition method. Mater. Sci. Eng. C.

[34] Castro-Cabado M., Casado A.L., San Román J. (2016). Bio-based thermosets: Effect of the structure of polycarboxylic acids on the thermal crosslinking of maltodextrins. Eur. Polym. J..

[35] Shi R., Bi J., Zhang Z., Zhu A., Chen D., Zhou X., Zhang L., Tian W. (2008). The effect of citric acid on the structural properties and cytotoxicity of the polyvinyl alcohol/starch films when molding at high temperature. Carbohydr. Polym..

[36] Xu H., Shen L., Xu L., Yang Y. (2015). Low-temperature crosslinking of proteins using non-toxic citric acid in neutral aqueous medium: Mechanism and kinetic study. Ind. Crops Prod..

[37] Lin H.M., Lin Y.H., Hsu F.Y. (2012). Preparation and characterization of mesoporous bioactive glass/polycaprolactone nanofibrous matrix for bone tissues engineering. J. Mater. Sci.: Mater. Med..

[38] Tian H., Xu G., Yang B., Guo G. (2011). Microstructure and mechanical properties of soy protein/agar blend films: Effect of composition and processing methods. J. Food Eng..

[39] Zhang M., Jiang W., Liu D., Wang J., Liu Y., Zhu Y., Zhu Y. (2016). Photodegradation of phenol via C3N4-agar hybrid hydrogel 3D photocatalysts with free separation. Appl. Catal. B.

[40] Liu Z., Ge X., Lu Y., Dong S., Zhao Y., Zeng M. (2012). Effects of chitosan molecular weight and degree of deacetylation on the properties of gelatine-based films. Food Hydrocoll..

[41] Etxabide A., Leceta I., Cabezudo S., Guerrero P., de la Caba K. (2016). Sustainable fish gelatin films: From food processing waste to compost. ACS Sustain. Chem. Eng..

[42] Rani G.U., Konreddy A.K., Mishra S. (2018). Novel hybrid biosorbents of agar: Swelling behaviour, heavy metal ions and dye removal efficacies. Int. J. Biol. Macromol..

[43] Raphael E., Avellaneda C.O., Manzolli B., Pawlicka A. (2010). Agar-based films for application as polymer electrolytes. Electrochim. Acta.

[44] Uranga J., Etxabide A., Guerrero P., de la Caba K. (2018). Development of active fish gelatin films with anthocyanins by compression molding. Food Hydrocoll..

[45] Li H., Liu B.L., Gao L.Z., Chen H.L. (2004). Studies on bullfrog skin collagen. Food Chem..

[46] Thakur V.K., Singha A.S. (2015). Surface Modification of Biopolymers.

